# Coagulation support algorithm with rapid TEG and functional fibrinogen TEG in critical bleeding: more results and less time

**DOI:** 10.1186/cc14432

**Published:** 2015-03-16

**Authors:** E De Blasio, C Pellegrini, A Federico, V Rocco, M Fumi, Y Pancione, S Sale, D Liberti

**Affiliations:** 1 Hospital G. Rummo, Benevento, Italy

## Introduction

**Figure 1:**
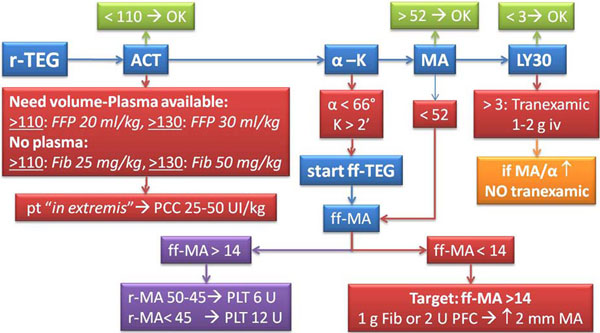
**Coagulation Support Algorithm**.

Early coagulation support is essential in massively bleeding patients. A Coagulation Support Algorithm (CSA), integrating rapid TEG (r-TEG) and functional fibrinogen TEG (ff-TEG) could shorten the time to a tailored treatment (Figure 1).

## Methods

A retrospective comparison of the time to available TEG and Standard Coagulation Tests (SCT: INR, aPTTr, fibrinogen level) results in two groups of bleeding and coagulopathic patients using citrate kaolin-TEG (k-TEG) or the CSA protocol (r-TEG/ff-TEG). Statistical analysis was performed with Student's *t *test for unpaired samples.

## Results

Twenty-three patients for each k-TEG and CSA group were compared. The time to available results was shorter using the CSA protocol in comparison with k-TEG (Table 1). The differences were both statistically (*P *< 0.00001) and clinically (mean reduction time 21 minutes) significant. SCT needed the longest time to obtain the final results.

**Table 1:** Comparison of time to results.

Test	k-TEG	r-TEG	r-TEG + ff-TEF	SCT
r (minutes)	13.8 ± 7.1	2.6 ± 2	-	
ACT (seconds)		265.7 ± 171.9	-	105.2 ± 46.3
TMA (minutes)	42.6 ± 12.4	25.4 ± 14.1	-	
CSAT (minutes)			21 ± 7.4	

Data presented as mean ± SD. ACT, activated clotting time; CSAT, Coagulation Support Algorithm total time; SCT, standard coagulation tests; TMA, time to maximum amplitude. r: r-TEG versus k-TEG, *P = *0.0000003; r-TEG versus SCT, *P = *0.000000001; k-TEG versus SCT, *P = *0.000000009; TMA: r-TEG versus k-TEG, *P = *0.0001; r-TEG versus SCT, *P = *0.00000004; k-TEG versus SCT, *P = *0.000002; ACT versus r of k-TEG (seconds), *P = *0.000004; CSAT versus k-TMA, *P = *0.00000005.

## Conclusion

The implementation of a CSA, including r-TEG and ff-TEG, could shorten the time to a targeted treatment in critically bleeding patients.
